# Surveillance strategies for Classical Swine Fever in wild boar – a comprehensive evaluation study to ensure powerful surveillance

**DOI:** 10.1038/srep43871

**Published:** 2017-03-07

**Authors:** Katja Schulz, Marisa Peyre, Christoph Staubach, Birgit Schauer, Jana Schulz, Clémentine Calba, Barbara Häsler, Franz J. Conraths

**Affiliations:** 1 Friedrich-Loeffler-Institut, Federal Research Institute for Animal Health, Institute of Epidemiology, Südufer 10, 17493 Greifswald–Insel Riems, Germany; 2 Centre de Coopération Internationale en Recherche Agronomique Pour le Développement (CIRAD), Département ES, UPR AGIRs, TA C22/E, Campus International de Baillarguet, 34398 Montpellier Cedex 5, France; 3 Royal Veterinary College, Veterinary Epidemiology, Economics and Public Health Group Hawkshead Lane, North Mymms, Hatfield AL9 7TA, UK

## Abstract

Surveillance of Classical Swine Fever (CSF) should not only focus on livestock, but must also include wild boar. To prevent disease transmission into commercial pig herds, it is therefore vital to have knowledge about the disease status in wild boar. In the present study, we performed a comprehensive evaluation of alternative surveillance strategies for Classical Swine Fever (CSF) in wild boar and compared them with the currently implemented conventional approach. The evaluation protocol was designed using the EVA tool, a decision support tool to help in the development of an economic and epidemiological evaluation protocol for surveillance. To evaluate the effectiveness of the surveillance strategies, we investigated their sensitivity and timeliness. Acceptability was analysed and finally, the cost-effectiveness of the surveillance strategies was determined. We developed 69 surveillance strategies for comparative evaluation between the existing approach and the novel proposed strategies. Sampling only within sub-adults resulted in a better acceptability and timeliness than the currently implemented strategy. Strategies that were completely based on passive surveillance performance did not achieve the desired detection probability of 95%. In conclusion, the results of the study suggest that risk-based approaches can be an option to design more effective CSF surveillance strategies in wild boar.

The continuous surveillance of animal diseases is an important element of animal health management and helps to minimize the risk of infectious diseases spreading through the international movement of animals and their products. Globalization and increased trade activities emphasize the need of reliable and powerful surveillance strategies^1^. To ensure that surveillance strategies result in the required certainty regarding the respective disease status, it is necessary to evaluate the strategies regularly. Moreover, only periodic evaluation allows identifying failures or inefficiencies and improving surveillance strategies when necessary.

Despite the obvious need to perform appropriate surveillance in animals, the funds available are limited^2^,^3^,^4^. In view of these constraints, surveillance should also be as cost-effective as possible.

Evaluation is critical to assess the quality of the data generated by these systems and to allow for priorisation of resource allocation, also in developed countries^5^. During the last fifteen years, extensive work has been conducted to define relevant attributes suitable for evaluation of the effectiveness of those systems^6^,^7^. Recently an integrated evaluation framework (the EVA tool) has been developed to provide a standardized way of implementing evaluation and assessing evaluation attributes and to promote economic evaluation^8^.

With regard to the surveillance objective of demonstrating disease freedom, Drewe *et al*.^9^ recommended analysing the evaluation attributes sensitivity and timeliness. In this context, the sensitivity of a surveillance strategy is defined as the probability of detecting an outbreak of the disease under surveillance^10^,^11^. The term timeliness describes the time between the introduction of an infection into the population and the time of detection^12^. The functionality of a system can be measured by using acceptability as an evaluation attribute^8^,^10^. Acceptability is defined as the willingness of a person or institution to contribute to the implementation of a strategy or system^13^.

To increase the effectiveness of surveillance, risk-based approaches can be taken into account. They can also be an option to save costs and to achieve the same or even better information, if risks have been identified and characterized^2^,^14^. According to the World Organisation for Animal Health^15^, risk is defined as the probability of the occurrence and the severity of the consequences of a health hazard for animal health. Hoinville *et al*.^1^ defined risk-based surveillance as the consideration of this risk, when designing or implementing surveillance strategies, but also when analysing surveillance data. Several studies recommended setting priorities on the basis of potential risk factors during the planning of animal disease surveillance^16^,^17^. However, a well-conducted risk assessment to ensure that the identified risk factors are truly associated with the higher probability of disease occurrence is a prerequisite to designing and applying risk-based surveillance efficiently^18^.

In the present study, a conventional surveillance strategy was compared with alternative, mainly risk-based, strategies. The analysis was done for Classical swine fever (CSF) in wild boar, which is susceptible to the disease and may form a virus reservoir. Therefore, it is important to demonstrate freedom from disease and achieve timely detection in this species because of the economic importance of CSF and a need to optimize the use of surveillance resources. The last outbreak of CSF in wild boar in Germany was recorded in 2009 in the federal state of Rhineland-Palatinate. Immunization with an attenuated live vaccine was used to control the disease in wild boar. The vaccination campaigns ended in 2012. Intensive surveillance during and after the vaccination campaigns, provided finally no longer evidence for the presence of CSF in the wild boar population. In June 2012, Germany therefore was declared as officially free from CSF. Thus, CSF is a suitable example to assess surveillance options to demonstrate freedom from disease with data of the German CSF surveillance system.

In Germany, active surveillance is currently conducted in accord with the Decision 2002/106/EG of the European Commission of 1 February 2002. It stipulates that the sample size must be large enough to detect a seroprevalence of 5% in the wild boar population with 95% confidence. To fulfil these requirements, at least 59 samples originating from healthy shot wild boar (active surveillance) need to be examined per year and district (“Landkreis”)^19^. The sample size calculations of Cannon and Roe^19^ assume an infinite population size, a homogeneous distribution of the infection in the population and a perfect diagnostic test. It is known, however, that several risk factors exist, which may increase the probability of wild boar contracting CSF. There are also factors, which can increase the probability of detecting CSF virus. On the basis of these considerations, alternative, mainly risk-based, surveillance strategies were developed.

To evaluate the current and potential alternative surveillance strategies, suitable evaluation attributes were selected and assessed to inform/provide recommendations.

The described approach may also be used as a template to evaluate surveillance strategies for other wild boar diseases.

## Materials and Methods

### Study areas and data

Data on wild boar and CSF surveillance were used from three federal states of Germany, namely Rhineland-Palatinate (RP), Mecklenburg-Western Pomerania (MV) and Lower Saxony (LS). These three federal states were chosen due to data suitability and availability. For the calculation of sensitivity and timeliness of the surveillance strategies, the districts of RP were used as the study area. Investigations employing participatory methods were conducted in RP and MV. Furthermore, data from all three federal states were used to parametrize the simulation model.

### Risk factor analysis

To identify risk factors for infection of wild boar with CSF virus or for detecting the virus in wild boar, a literature search was conducted and potential risk factors were analysed for potential associations with virological or serological test results regarding CSF.

#### Literature analysis

The databases Web of Science^TM^ Core Collection, CABI: CAB Abstracts^®^ and Global Health^®^ were searched. Only publications that appeared in English or German from 1990 through to 2014 were taken into account. The following search term was used: ((risk* OR incidence* OR prevalence*) AND (“wild boar” OR “wild pigs” OR “feral pigs”) AND (“ classical swine fever”)). Parts of the identified factors were included in a statistical analysis.

#### Statistical analysis

Statistical analyses were done using the statistical software R (http://www.r-project.org).

Potential risk factors identified by the systematic literature search were tested for statistically significant associations with serological and virological examination results for CSF using Pearson’s Chi-squared test^20^. This was done on the basis of infection data of MV. A p-value of <0.05 was considered as statistically significant.

The data of MV had been collected between 1993 and 2000, when CSF was endemic in the wild boar population in this federal state. A total of 172,906 data records were available and information on the variables date, location, age and serological and virological results were used. The data set is described in detail by Schulz *et al*.^21^.

Only the risk factors age and season were tested statistically. The risk factors population density and samples originating from passive surveillance, identified through the literature search could not be tested due to a lack of suitable data.

To test the null hypothesis, i.e. the number of serologically or virologically positive animals is equally distributed in each age class (piglets: <1 year; sub-adults: 1–2 years; adults >1 year) for each case, two age classes were summarized and tested against the third. To investigate the influence of season on the test results, a categorical variable “season” was generated. Results obtained in the months October until March were categorized as 1 and those from the remaining months as 2.

### Evaluation protocol

The EVA tool was used to define the integrated epidemiological and economic evaluation protocol. Briefly, information on the surveillance system and components under evaluation were imputed into the RISKSUR online web tool (http://webtools.fp7-risksur.eu), along with the aim and context of the evaluation. This allowed selecting the most appropriate evaluation questions adapted to the context and the objectives of the study. The tool generated a suitable selection of evaluation attributes and measurement methods for assessing the efficiency, effectiveness, functional aspects influencing the overall performance of a surveillance system and economic criteria. The evaluators ranked the evaluation attributes based on assessment methods and data availability to identify the ones to be assessed within this evaluation.

### Sensitivity and Timeliness

**Figure 1:**
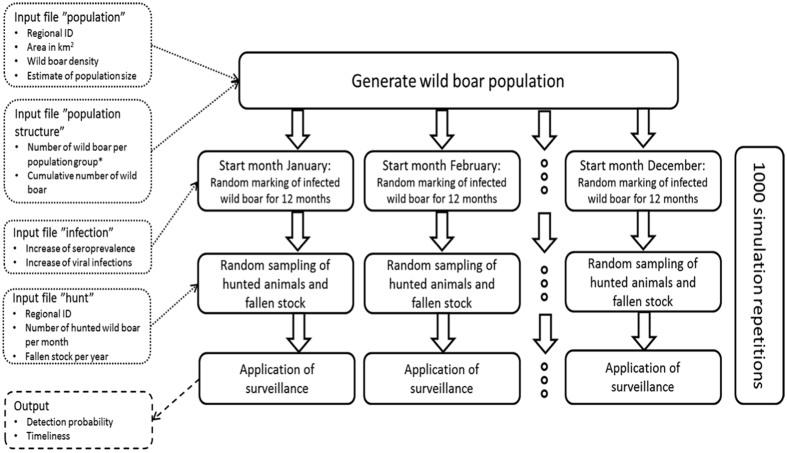
Structure of the simulation model to calculate sensitivity and timeliness of the current and alternative surveillance strategies for Classical Swine Fever in wild boar in Germany (adapted from Schulz *et al*.^21^). *Population group: number of individuals in each of the combinations of age classes, gender and cause of death.

A simulation model was used to determine the sensitivity and timeliness of different surveillance strategies for CSF in wild boar. To simulate the different surveillance strategies and to calculate their performance, real surveillance data was used to prepare the input files for the model. The evaluation attributes sensitivity and timeliness were calculated as output variables. The structure of this model is described in detail by Schulz, *et al*.^21^. In the present study, different data for the input files were used. In the following text only the differences to the initial model description of Schulz *et al*.^21^ are outlined. Figure 1 is adapted from Schulz *et al*.^21^ and illustrates the model structure in detail.

All analyses were done in R (www.r-project.org). An example of the source code of the R script is provided as Supplementary Information and the complete R script can be obtained from Jana Schulz.

First, a wild boar population was generated (input file “population”) and an appropriate number of wild boar was allocated to each district (“Landkreis”) of the federal state of RP (Fig. 1).

The variables age, gender and cause of death (hunted, shot sick, found dead, road traffic accident (RTA)) were assigned to each animal in the population generated in the model, depending on the appropriate proportion in a real data set of RP (Fig. 1), (input file “population structure”, Table 1).

To simulate a seroprevalence of 5%, a proportion of 5% of the animals were marked as serologically positive in the start month. The detailed age and sex distribution of the animals marked as infected is described in Schulz *et al*.^21^ (Fig. 1). The serological and virological increase in the following months was simulated by using real infection data (input file “infection”).

Out of the generated population, wild boar were randomly marked as hunted or detected through passive surveillance (shot sick, found dead or RTA) (Fig. 1). The proportion of hunted animals per month was calculated on the basis of real hunting data (input file “hunt”).

To simulate surveillance, 59 samples (number of samples depending on the particular surveillance strategy (Tables 2)) were randomly chosen out of the animals marked as hunted. In contrast to the study of Schulz *et al*.^21^ (Fig. 1), to simulate as realistic as possible, the temporal distribution of sampling was not only simulated randomly but also based on real data (input file “surveillance”).

For the surveillance strategies, simulations were conducted, in which samples were either examined only serologically, only virologically or by both methods. To estimate the sensitivity and timeliness of surveillance strategies, the results of 1,000 simulation runs were used per scenario (Fig. 1).

To compare the calculated detection probability for each surveillance strategy, the arithmetic mean of the detection probabilities for all starting months of infection were calculated. This led to one value for each surveillance strategy. To assess timeliness, we calculated the percentage of simulation runs, which detected the infection in each month after the start of infection. We determined the average value of these calculations for each following month and weighted early detection. This resulted in a comparable value for the timeliness of the investigated surveillance strategy. A more detailed description of the calculation of the two evaluation attributes can be found in Schulz *et al*.^21^.

#### Data background

##### Input file “population”

Data obtained from studies of the Research Institute for Forest Ecology and Forestry of the federal state of Rhineland-Palatinate (RP), Trippstadt, Germany from 2012 were used. The data on the wild boar population was determined using feces and tissue samples^22^. The data set contained the estimated number of wild boar for all 36 districts of RP. However, data from 12 urban districts were excluded as the number of wild boar in these districts was negligible.

##### Input file “population structure”

Surveillance data from three different areas of RP with information on the variables age, sex and cause of death were used. The data originated from the European CSF databank of the Friedrich-Loeffler-Institut (CSF-Database; http://public.csf-wildboar.eu/Default.aspx).

In total, 120,134 data sets were available. After removing all data records that did not contain information on age, sex or cause of death, 105,439 data records were available, which covered the time period from 15 March 2003 through to 22 July 2014.

##### Input file “Infection”

Data originating from MV were used to estimate the seroprevalence and the virological CSF prevalence in the simulated infected area. Data and processing have been described by Schulz *et al*.^21^.

##### Input file “Hunt”

Hunting data provided by the Supreme Hunting Authority of the Ministry of Environment, Forestry and Consumer protection of RP were used to generate the input file “hunt”. A detailed description of the data can be found in Schulz *et al*.^21^.

##### Input file “Surveillance”

To simulate the distribution of sampling over the year based on real data, data of the State Office of Consumer Protection and Food Safety of the federal state of Lower Saxony, Oldenburg, Germany, was used. The dataset consisted of 12,784 data records, originating from 37 districts and included data from 2011 through to 2014. It contained information about the location and the date of sampling, the age of the sampled animal and on the surveillance type (i.e. active or passive surveillance). After excluding records with incomplete information on these variables, 12,620 data records were available for the model. The data of the 37 districts were summarized and averaged on a monthly basis. Depending on the age and origin of the sampled animals, the percentage of sampled wild boar per month was calculated.

#### Sensitivity analysis

A sensitivity analysis was performed as described in Schulz *et al*.^21^.

### Acceptability

To investigate the acceptability of the current surveillance system by hunters, participatory methods, developed by Calba *et al*.^23^ (AccEPT) were used. By conducting eight focus group discussions with hunters from RP and MV, the trust in the system, the acceptability of the operations and of the objectives of the system were investigated. Additionally, five surveillance strategies, developed on the basis of the risk factor analyses were presented to the hunters and the acceptability of these strategies investigated using methods of participatory epidemiology as described in the study of Schulz *et al*.^24^.

### Cost-effectiveness

For all strategies for which the simulation model resulted in a detection probability of at least 95% and thus met the requirement of the EU regulations, cost estimates were obtained and the differences to the costs of the reference strategy (Table 2) calculated. The cost analyses were done in Excel (Microsoft version 2010). Transport costs and costs for the examination of samples were included in the calculations as described below. Other costs were not included as deemed the same across the different strategies.

#### Transport costs

Transport costs consisted of fuel costs for the hunters, which occurred when they drove to the hunting ground and back to the respective place (district veterinary office) to deliver the wild boar sample. A distance of 100 km per trip with a fuel consumption of 10 l/100 km was assumed. 1.5 Euro/l was specified; therefore costs of 15 Euro per ride were assumed. Secondly, the costs for the system, which comprised the fuel cost of an official driver of the central laboratory, to which the samples were submitted, were included. The laboratory driver had to pick up the samples from the district veterinary office and bring it to the laboratory. It was assumed that the laboratory driver does 10 rides per month and has to cover 100 km per trip. Therefore, the transport costs for the system were assumed to be 150 Euro per month.

For each surveillance strategy, the calculated costs for the hunters and the system were summarized.

#### Laboratory testing costs

It was differentiated between costs for serological and virological examinations. Costs for serology were estimated as 3.00 Euro per sample and costs for virological tests as 9.90 Euro. The examination costs for one year were summed up depending on the number and the kind of performed examinations.

The total costs were summed up and multiplied by 24 to account for the 24 districts included in the study (12 municipal districts excluded). For some newly developed surveillance strategies limiting surveillance to districts with a defined wild boar density, the multiplication factor was accordingly smaller.

### Overall evaluation

Due to the large number of developed surveillance strategies, it was not feasible to investigate each evaluation attribute for all strategies. Therefore, the comparison of the performance of the strategies was done in groups. Each surveillance strategy was assigned to a group depending on the evaluation attributes, which were examined for the corresponding strategy. Consequently, the following four groups were created:

Group 1: Strategies with a detection probability of at least 95% and for which acceptability was investigated (sensitivity, timeliness, acceptability and cost-effectiveness).

Group 2: Strategies with a detection probability of at least 95% without taking acceptability into account (sensitivity, timeliness and cost-effectiveness).

Group 3: Strategies, independently of costs, for which acceptability was investigated (sensitivity, timeliness and acceptability).

Group 4: Strategies, independently of costs, without taking acceptability into account (sensitivity and timeliness).

In each group, the strategies were ranked according to the estimates obtained for the evaluation attributes. The ranking was ascending, i.e. the best result was assigned a value of 1. Since the sensitivity values were very similar, all strategies with a detection probability of 95% and above were assigned a rank of 1, whereas all strategies with a detection probability below 95% received a rank of 2. This approach helped also to deal with potential fluctuations of the simulation model. For each strategy, the arithmetic mean of the assigned ranks was calculated and the surveillance strategies ranked in each group according to their overall performance.

### Ethical approval

In the focus group discussions, all hunters participated voluntarily and were informed about the objectives of the study. Formal consent or ethics approval was not required for this study as it did neither include clinical trials in humans, where ethical approval would have been required according to paragraph 40 of the German “Arzneimittelgesetz” or paragraph 20 of the German “Medizinproduktegesetz” nor animal experiments, where ethical approval would have been required according to paragraph 15 of the German “Tierschutzgesetz” (Animal Health Act).

## Results

### Risk-factor analysis

Age, population density, season and samples originating from passive surveillance were through the literature search identified as risk factors for an infection of wild boar with CSF virus or for detecting the virus in wild boar samples. Age and season were confirmed as statistically significant. Based on the results of the risk factor analysis, 31 different strategies were developed (Tables 2, 3, 4). Strategy 1 (Table 2) represents the currently implemented surveillance strategy and was used as reference strategy in this analysis. As for some strategies it was simulated that samples were not only examined serologically or virologically but also by both methods, the performance of a total of 69 different surveillance strategies was investigated.

### Evaluation protocol

The selected evaluation questions were: 1) to assess the costs and effectiveness of surveillance components (out of two or more) to determine which achieves a defined effectiveness target at least cost, the effectiveness needs to be determined; 2) to assess the effectiveness of one or more surveillance components and the functional aspects of surveillance that may influence the effectiveness. Based on the tool recommendations and evaluator ranking, 4 attributes were selected for assessment (Table 5):

-Sensitivity

-Timeliness

-Acceptability

-Costs to run the cost-effectiveness analysis and optimize surveillance efficiency.

### Sensitivity and Timeliness

Sampling was simulated as random sampling. Furthermore, sampling was simulated based on real data in all developed strategies (input file “surveillance”).

**Figure 2:**
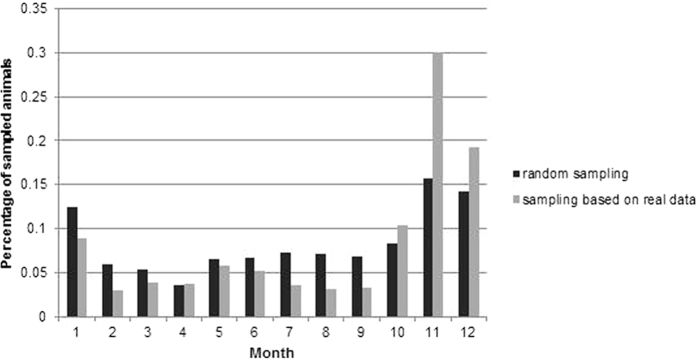
Percentage of samples per month obtained by a simulation of random sampling (black columns) or a simulation based on real data (grey columns).

Simulating the sampling based on real data showed seasonality. In the months of November and December a larger proportion of samples was taken then in the other months. Sampling randomly throughout the year showed the same tendency, but the seasonal effect was less pronounced (Fig. 2).

#### Sensitivity

The detection probability of the strategies did not differ significantly between the simulation of random sampling throughout the year and the simulation of sampling on the basis of real data (data not shown). Therefore, only the results of the temporal distribution of sampling simulated using real data are shown in the following sections.

**Figure 3:**
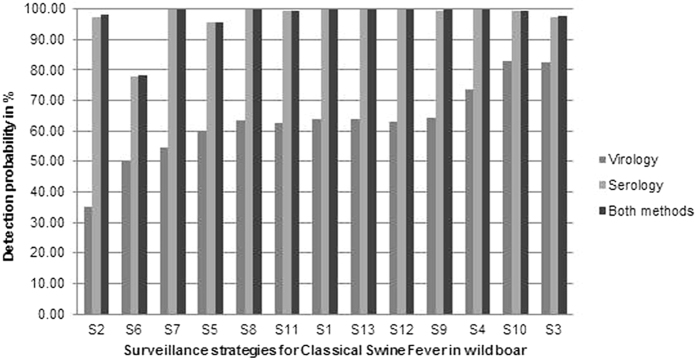
Comparison of the detection probabilities of the strategies, for which samples were simulated to be examined serologically, virologically or by both methods. Numbers on the x-axis refer to the numbers of strategies in Tables 2, 3, 4.

Simulating the simultaneous serological and virological examination of samples didn’t improve the detection probability significantly compared to the simulation of an exclusive serological examination. However, simulating only the virological examination of samples resulted in a substantially lower detection probability (Fig. 3).

Strategies (S), in which samples were simulated to be examined only serologically, showed an averaged detection probability of 96.55% (Table 6). The detection probability was lowest for S 18 (10, Table 2) with 77.32% and highest for S 28 (all passive + 50) with 99.97%.

The highest detection probability achieved by strategies, for which only a virological investigation was simulated, was 83%. Strategy 25 (50% passive, Table 3) showed with nearly 21% the lowest and strategy 10 (120 NDJ, Table 2) with 82.94% the highest value.

The averaged detection probability of the strategies, for which had been simulated that samples were investigated by both serology and virology, showed with 97.20% a slightly higher value then investigating the samples only serologically (Table 6). Strategy 1 (59, Table 2), which is the currently implemented surveillance strategy, showed with almost 100% the best detection probability, whereas strategy 6 (59 district >4, Table 2) yielded with 78.07% in the lowest detection probability.

#### Timeliness

The resulting values for timeliness did not significantly differ between the simulations of random sampling and the sampling based on real data.

**Figure 4:**
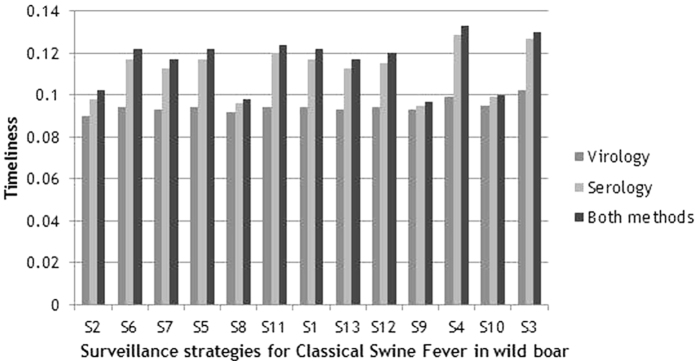
Comparison of timeliness of the strategies, for which samples were simulated to be examined serologically, virologically or by both methods. Numbers on the x-axis refer to the numbers of strategies in Tables 2, 3, 4.

Also, the difference between investigating the samples only serologically, or serologically and virologically was small (Fig. 4). However, the application of both test methods always resulted in a slightly higher value for timeliness. For all strategies, in which samples were modelled to be examined only virologically, timeliness displayed lower values (Table 6).

For the strategies, in which samples were only investigated serologically in the model, S 4 (59 sub-adults, Table 2) showed the highest value (0.129) (Table 6). The lowest timeliness (0.079) was obtained for S 32 (all passive +10, Table 4).

Strategy 3 (59 adults, Table 2) resulted in a timeliness of 0.102 and hence showed the highest value for the strategies, in which samples were only investigated virologically in the model. The timeliness of S 2 (59 piglets) was 0.090 (Table 6).

The strategies, in which samples were investigated by both methods in the model, resulted in an average timeliness of 0.113. S 18 (10, Table 2) showed the lowest value, whereas S 4 (59 sub-adults) yielded the highest score (Table 6).

#### Sensitivity analysis

Sensitivity analyses showed that the model was stable. For details, see Schulz *et al*.^21^.

### Acceptability

Four of the developed surveillance strategies were chosen (Table 7) and presented to the hunters together with the reference strategy to investigate the acceptability of these strategies.

Detailed results are described in Schulz *et al*.^24^. Briefly, strategy 4 was the most accepted strategy, whereas strategy 24 resulted in a very low level of acceptability. The reference strategy resulted in a score, which was just below the acceptability level of strategy 4. Strategies 11 and 27 were moderately accepted.

### Cost-effectiveness

Costs were calculated for 47 of the 69 surveillance strategies as they reached a detection probability of at least 95% in the simulation model. The costs of the conventional surveillance strategy for CSF in wild boar were used as a reference. Based on transport costs of 15 Euro per sample for the hunter, taking 59 samples per year resulted in 885 Euro. For the system, the assumed transport costs of 150 Euro per month resulted in yearly costs of 1,800 Euro. Accordingly the summarized transport costs for the reference strategy amounted to 2,685 Euro. The examination costs of 59 samples by serology amounted to 177 Euro.

Accordingly, the total costs for strategy 1 were 2,862 Euro per district and 68,688 Euro for all 24 districts (12 municipal districts excluded).

Among the strategies, for which only serological tests were simulated, strategies 8 and 9 (Table 2) were with only 36,288 Euro least expensive, while strategy 26 (Table 4) caused the highest costs exceeding the costs of the reference strategy by 7,171 €. When serological and virological examinations were modelled, the results stayed in the same range, except for the fact that the costs increased by the expenditures for the virological tests.

### Overall evaluation

Within group 1, strategy 4 (Table 2), where at least 59 samples were only taken in the age class of sub-adults, resulted in the highest overall score, while strategy 13 yielded the lowest score (Table 8).

In group 2, where acceptability was not included, quarterly sampling (strategies 11, 12 and 13) resulted in the highest overall score. In the two remaining groups, strategy 4 (Table 2) showed, like in group 1, the best overall performance (data not shown). Strategies, where only 10 samples were taken (strategies 18 and 32), resulted generally in low scores. Due to low detection probability and bad acceptability, strategies 24 and 25 (Table 3) also yielded low scores.

## Discussion

This study identified age, population density, season and animals, found through passive surveillance as risk factors for an infection of wild boar with CSF virus or for detecting the virus in wild boar samples, which is in agreement with the literature^25^,^26^,^27^,^28^,^29^. Based on the risk factor analysis, alternative surveillance strategies for CSF in wild boar in Germany were developed. The majority of these strategies were risk based, i.e. the sampling took place only in a defined age class with an increased risk of infection, or only in areas with a wild boar density above a certain threshold. The objective of the study was to compare the performance of these surveillance strategies with the currently implemented strategy in Germany as the reference and to identify more effective strategies using this approach.

Three evaluation attributes (sensitivity, timeliness and acceptability) were examined and an economic evaluation was performed. The design of the evaluation protocol using the EVA tool allowed targeting the evaluation to meet the specific requirement of the decision makers. An evaluation process can be time and resource consuming and should therefore be framed appropriately. The EVA tool not only helped in identifying the most relevant economic evaluation questions and methods to implement but also highlighted critical aspect such as acceptability of the surveillance to be considered in order to ensure its efficacy. Other attributes were highlighted as relevant to assess within this evaluation process, but only a reduced number of attributes were included to compare the performances of the different surveillance developed in this study. This was based partly in order to limit the resources involved in the evaluation process, but also based on practical issues such as data and/or method availability. If none of the considered attributes had allowed discrimination between the strategies, additional attributes would have been included as recommended in the EVA tool instructions. Another advantage of using the EVA tool was in the feedback and documentation of the evaluation process and results to decision makers. This evaluation framework promotes comprehensive and economic evaluation based on published literature and expert opinion. It also provides relevant information to justify the different choices made to frame the evaluation and to ensure transparency and appropriate documentation of the whole process.

Sensitivity and timeliness were examined using a simulation model. The advantage of this method is the possibility to simulate surveillance and the detection of the first case in times of disease freedom. Since real data was used as input, the model may come close to reality and uncertainties can be minimized.

When comparing the sensitivity and timeliness of surveillance strategies with respect to the distribution of sampling over the year, it was found that the results of the majority of strategies did not differ considerably. This can be explained by the seasonality of hunting. Also, when sampling was simulated randomly throughout the year, only hunted animals could be sampled. During winter, the number of hunted wild boar is much higher as compared to other seasons^25^,^30^. Accordingly, the seasonality in sampling observed when sampling was simulated based on real data, could also be seen in the simulation of random sampling. S 25 (50% passive, Table 3) resulted in a lower detection probability and timeliness when sampling was simulated as a random process. This may be due to the low number of available data for this scenario.

As already reported in another study^21^, samples examined not only serologically but serologically and virologically in the model failed to lead to a significant improvement of the detection probability and timeliness of various surveillance strategies. It can thus be concluded that in times of disease freedom it would be sufficient to examine the samples only by serology as this saves resources.

It is known that the probability to detect CSF antibodies is higher in older animals^25^,^31^. We therefore developed strategies, in which samples are only taken in certain age classes. The results showed that for example sampling only the class of sub-adult animals (1–2 years) yielded a similar detection probability as the reference strategy. However, due to its higher timeliness, risk-based sampling may still be advantageous as postulated by Stärk *et al*.^2^ and Viennet *et al*.^32^. It may even justify a reduction of the sample size.

S 5 (59 district >2) and S 7 (sample size/population, Table 2), which were simulated with regard to the risk factor ‘population density’ showed useful results. S 5 (59 district >2) was developed on the basis of a publication describing that a population density of >2 wild boar per square kilometre carries an increased risk of virus spread^33^. However, the determination of true population sizes in wildlife is difficult^34^.

The low timeliness in strategies, where sampling was only done during the hunting season, was expected, as the infection can only be detected if sampling takes place. Failure of sampling in some months therefore leads to late detection of infections, i.e. low timeliness. Accordingly, implementing such strategies would bear the risk of detecting the infection months after the introduction of CSF virus. It has to be mentioned, however, that the result of a CSF outbreak would be increased numbers of dead wild boar^27^. It is therefore likely that such an outbreak would be detected earlier by passive surveillance, even in the absence of active surveillance.

The acceptability by hunters of different surveillance strategies for CSF in wild boar was investigated by participatory methods. In contrast to common scientific methods, these methods, which were originally developed in social sciences, are mainly based on qualitative analyses^35^. This holds the risk of biased results^24^. Despite all limitations, including the measurement of acceptability in the evaluation of several surveillance strategies for CSF in wild boar, participatory methods yielded interesting results. Strategy 24 (Table 3) was hardly accepted by the hunters. The probability that an animal is CSF-infected when found dead or sick, is increased^25^,^27^,^29^. However, sampling only wild boar found through passive surveillance would not be feasible as the key players fail to support such an approach. In contrast, also with regard to acceptability, a risk-based surveillance strategy, namely sampling only the age-class of sub-adults, achieved the best results. The hunters argued that sampling only wild boar of 1–2 years of age was easier for them as these animals are more frequently shot anyway. Accordingly, such a strategy would be well accepted and implementing it may be promising.

The calculated costs only referred to costs expected in times of disease freedom. The assumptions, under which the calculations were performed, should be checked in reality for the respective situation. However, as the costs for each strategy were calculated under the same assumptions and as only cost relations were taken into account, the results may be useful, in particular for comparing different scenarios.

The overall assessment was done in groups, as it was not possible to investigate each evaluation attribute in all the surveillance strategies. The economic evaluation was only done for those strategies, which reached a detection probability of 95% and therefore an effectiveness of 100%.

Except for the results in group 2, where acceptability was not included in the analyses, the strategy, in which at least 59 samples were only taken from the age-class of sub-adults (strategy 4, Table 2), showed the best overall performance. This result corresponds with results of previous studies, in which it was shown that the probability of detecting serologically positive animals is higher in older than in young animals^31^,^36^. Sampling only in the age-class of adult animals resulted in lower values for timeliness. This may be explained by the smaller proportion of animals of this age class in the hunted population (Table 1). This is in turn likely to be the consequence of the current hunting recommendations, which propose to shoot mainly young and sub-adult wild boar.

Despite the low costs, strategies that include the risk factor seasonality (strategies 8 and 9, Table 2) failed to perform better than the reference strategy. This was mainly due to the low value for timeliness. Implementing such strategies should therefore be critically reflected.

Sampling only in areas with a wild boar density above a defined threshold (strategies 5 and 6) or adapting the sample size based on the wild boar density (strategy 7, Table 2) resulted for strategies 5 and 7 in an acceptable performance. They were less expensive than the reference strategy, while sensitivity and timeliness showed comparable values. However, due to the difficulties concerning the determination of precise population estimates, the implementation of these strategies is challenging. Instead of using the population density as a threshold, the sample size could be chosen depending on the size of the hunting bag of the respective districts.

The results in group 2 emphasize the importance of the inclusion of more evaluation attributes. Also Drewe *et al*.^10^ recommended including several attributes in the evaluation of surveillance systems. If acceptability was not taken into account, strategies 11, 12 and 13 (Table 2) showed the best results. However, the hunters refused to accept these strategies. Trying to implement them, which may be tempting because of their low costs, high detection probability and good timeliness, without considering the low acceptability of the key-players, may be extremely risky and could cause the failure of CSF surveillance in wild boar. In addition, our results support the hypothesis that valuable information for the evaluation of surveillance can be obtained by methods of participatory epidemiology. This is consistent with results of other studies, in which used participatory methods were used^37^,^38^.

Taking only 50 or 40 samples instead of the minimum number of 59 required in the reference strategy also yielded good results. These strategies were not presented to the hunters; however, as their workload would be reduced, it can be assumed that the level of acceptability will be high. A decreased sample size would lead to lower costs, but the authorities in charge would need to ensure that the sampling is spatially and temporarily equally distributed^39^. It is likely that this increases the workload at another level, namely for the staff in the veterinary authorities. Participatory methods may also help to evaluate the acceptability of such a proposal by the local veterinary authorities. Increasing the sample size during the hunting season (strategy 10, Table 2), resulted in extremely high costs, so that even the slightly higher detection probability may not justify the implementation of such a surveillance strategy.

According to the results of the present study, the following conclusions may be drawn:

1. Risk-based strategies (sampling in the age-class of sub-adults) for CSF surveillance in wild boar constitutes an opportunity to improve the performance of surveillance strategies. This complies with previous studies^2^,^40^.
2. It may be reasonable to review the samples sizes calculated on the basis of Cannon and Roe^19^ with the aim of reducing the number of samples by limiting sampling to sub-populations with an increased risk of CSF infection or higher detection probability.
3. The currently implemented surveillance strategy for CSF in wild boar achieved satisfying results.

The present study revealed interesting alternatives to the currently implemented surveillance strategy for CSF in wild boar. It may also be considered as a template to evaluate other animal health surveillance systems.

## Additional Information

**How to cite this article:** Schulz, K. *et al*. Surveillance strategies for Classical Swine Fever in wild boar – a comprehensive evaluation study to ensure powerful surveillance. *Sci. Rep.* **7**, 43871; doi: 10.1038/srep43871 (2017).

**Publisher's note:** Springer Nature remains neutral with regard to jurisdictional claims in published maps and institutional affiliations.

## Supplementary Material

Supplementary Information

## Figures and Tables

**Table 1:** Population structure (number of wild boar) in the simulation model regarding age and sex of shot healthy (active surveillance), found dead, shot sick and wild boar, which were involved in road traffic accident (passive surveillance).

*Age	**Gender	***Surveillance type and cause of death	Number of wild boar	Percentage of wild boar	Number of wild boar in the simulation model
1	f	active	27,784	26.35%	24,768
		passive_dead	21	0.02%	19
		passive_sick	9	0.01%	8
		passive_RTA	74	0.07%	66
	m	active	29,236	27.73%	26,063
		passive_dead	17	0.02%	15
		passive_sick	8	0.01%	7
		passive_RTA	84	0.08%	75
2	f	active	17,375	16.48%	15,489
		passive_dead	16	0.02%	14
		passive_sick	5	0.00%	4
		passive_RTA	55	0.05%	49
	m	active	19,046	18.06%	16,979
		passive_dead	4	0.00%	4
		passive_sick	3	0.00%	3
		passive_RTA	48	0.05%	43
3	f	active	4,671	4.43%	4,164
		passive_dead	13	0.01%	12
		passive_sick	1	0.00%	1
		passive_RTA	23	0.02%	21
	m	active	6,914	6.56%	6,164
		passive_dead	5	0.00%	4
		passive_sick	2	0.00%	2
		passive_RTA	25	0.02%	22
**Total**			105,439		93,994

*1: 0–1 year, 2: 1–2 years, 3: >2 years, **f: female, m: male, ***active: shot healthy; passive_dead: found dead; passive_sick: shot sick; passive_RTA: road traffic accident.

**Table 2:** Active surveillance strategies; abbreviations in brackets, the surveillance strategy marked in bold is the currently implemented strategy and represents the reference strategy.

1*	**59 samples per district within one year, taken out of the whole hunting bag (59)**
2*	59 piglet samples per district, within one year (59 piglets)
3*	59 adult samples per district, within one year (59 adults)
4*	59 sub-adult samples per district, within one year (59 sub-adults)
5*	59 samples per district with a wild boar density >2.0 wild boar per km^2^, within one year, taken out of the whole hunting bag (59 district >2)
6*	59 samples per district with a wild boar density >4.0 wild boar per km^2^, within one year, taken out of the whole hunting bag (59 district >4)
7^2^*	Sample size per district depended on the population density (sample size/population)
8*	59 samples per district, sampled only in the main hunting season, in the months of November, December and January (59 NDJ)
9*	59 sub-adult samples per district, sampled only in the main hunting season, in the months of November, December and January (59 sub-adults NDJ)
10*	120 samples per district, sampled only in the main hunting season, in the months of November, December and January (120 NDJ)
11*	59 samples per district, sampled quarterly: January, April, July, October (JAJO)
12*	59 samples per district, sampled quarterly: February, May, August, November (FMAN)
13*	59 samples per district, sampled quarterly: March, June, September, December (MJSD)
14**	50 samples per district within one year, taken out of the whole hunting bag (50)
15**	40 samples per district within one year, taken out of the whole hunting bag (40)
16**	30 samples per district within one year, taken out of the whole hunting bag (30)
17**	20 samples per district within one year, taken out of the whole hunting bag (20)
18**	10 samples per district within one year, taken out of the whole hunting bag (10)
19**	50 sub-adult samples per district, within one year (50 sub-adults)
20**	40 sub-adult samples per district, within one year (40 sub-adults)
21**	30 sub-adult samples per district, within one year (30 sub-adults)
22**	20 sub-adult samples per district, within one year (20 sub-adults)
23**	10 sub-adult samples per district, within one year (10 sub-adults)

*Samples were investigated only serologically, only virologically or using both methods. **Samples were investigated only serologically or using both methods. ^2^Calculated with software developed by Dr. Andreas Fröhlich, Friedrich-Loeffler-Institut, Greifswald – Insel Riems, Germany (unpublished).

**Table 3:** Passive surveillance strategies; abbreviations in brackets.

24*	**Sampling of all wild boar found dead, shot sick or involved in road traffic accidents (all passive)**
25*	Sampling 50% of wild boar found dead, shot sick or involved in road traffic accidents (50% passive)

*In the scenarios, all samples were modelled to be investigated only virologically.

**Table 4:** Combined strategies of active and passive surveillance; abbreviations in brackets.

26*	**Sampling of all wild boar found dead, shot sick or involved in road traffic accidents +59 samples per district within one year, taken out of the whole hunting bag (all passive +59)**
27**	Sampling 50% of wild boar found dead, shot sick or involved in road traffic accidents +59 samples per district within one year, taken out of the whole hunting bag (50% passive +59)
28**	Sampling of all wild boar found dead, shot sick or involved in road traffic accidents +50 samples per district within one year, taken out of the whole hunting bag (all passive +50)
29**	Sampling of all wild boar found dead, shot sick or involved in road traffic accidents +40 samples per district within one year, taken out of the whole hunting bag (all passive +40)
30**	Sampling of all wild boar found dead, shot sick or involved in road traffic accidents +30 samples per district within one year, taken out of the whole hunting bag (all passive +30)
31**	Sampling of all wild boar found dead, shot sick or involved in road traffic accidents +20 samples per district within one year, taken out of the whole hunting bag (all passive +20)
32**	Sampling of all wild boar found dead, shot sick or involved in road traffic accidents +10 samples per district within one year, taken out of the whole hunting bag (all passive +10)

*All samples resulting from passive surveillance were modelled to be investigated virologically and samples resulting from active surveillance were modelled to be investigated only serologically or by both methods. **In the scenarios, all passive samples were modelled to be investigated virologically and all active samples were modelled to be investigated serologically.

**Table 5:** List of evaluation attributes generated by the EVA tool to evaluate the CSF surveillance system in wild boar and ranked by the evaluators according to assessment methods availability and data access.

Evaluation attribute		Assessment methods and tools	Data availability	Rank
Effectiveness	Sensitivity; Timeliness	Simulation modelling	Yes	1
	Negative predictive value; Bias and Representativeness	Capture Recapture	No	2
Functional	Acceptability and engagement	AccEPT	Yes	1
	Availability, sustainability			2
Economic	Cost	Cost analysis	Yes	1

**Table 6:** Maximum, minimum and averaged detection probability in %/**timeliness** of surveillance strategies, in which samples were modelled to be investigated only serologically, only virologically or by both methods.

	Diagnostic method		
	Serological	Virological	Both
Mean	96.55/**0.108**	57.37/**0.095**	97.20/**0.113**
Maximum	99.97/**0.129**	82.94/**0.102**	99.99/**0.133**
Minimum	77.32/**0.079**	20.79/**0.090**	78.07/**0.083**

**Table 7:** Strategies for CSF surveillance in wild boar, for which the acceptability by hunters was evaluated.

**1**	**Currently implemented** (**strategy 1**) 59 samples per district within one year, taken out of the whole hunting bag
**2**	**Passive** (**strategy 24**) Sampling of all wild boar found dead, shot sick or involved in road traffic accidents
**3**	**Quarterly** (**strategy 11**, **12**, **13**) 59 samples per district, sampled quarterly
**4**	**Sub-adults** (**strategy 4**) 59 sub-adult samples per district, within one year
**5**	**Strategy 1 combined with 50% passive** (**strategy 27**) Sampling 50% of wild boar found dead, shot sick or involved in road traffic accidents +59 samples per district within one year, taken out of the whole hunting bag

The numbering of the strategies in brackets refers to that in Tables 2, 3, 4.

**Table 8:** Analyses of group 1: Overall evaluation of all strategies, in which all three evaluation attributes and costs were investigated.

Strategy	Sensitivity in %	S	Timeliness	S	Acceptability	S	Cost difference in Euro	S	Total score
S4 sero	99.76	1	0.129	2	1	1	0	3	1.8
S4 vise	99.82	1	0.133	1	1	1	14,018.4	5	2.0
S11 vise	99.47	1	0.124	3	−0.4	4	−27,146.4	2	2.5
S11 sero	99.27	1	0.12	5	−0.4	4	−28,800	1	2.8
S1 vise	99.99	1	0.122	4	0.9	2	14,018.4	5	3.0
S1 sero	99.95	1	0.117	6	0.9	2	0	3	3.0
S12 vise	99.91	1	0.12	5	−0.4	4	−27,146.4	2	3.0
S13 vise	99.82	1	0.117	6	−0.4	4	−27,146.4	2	3.3
S12 sero	99.82	1	0.115	8	−0.4	4	−28,800	1	3.5
S27 sero	99.96	1	0.116	7	−0.3	3	3,585.6	4	3.8
S13 sero	99.72	1	0.113	9	−0.4	4	−28,800	1	3.8

S represents the score, whereby 1 constitutes the best result; sero = simulation of serological sample examination, vise = serological and virological sample examination.
