# Supplementary material for: Surveillance strategies for Classical Swine Fever in wild boar – a comprehensive evaluation study to ensure powerful surveillance
Source: Sci Rep. 2017 Mar 7;7:43871. doi: 10.1038/srep43871 (PMC5339697; doi:10.1038/srep43871)
Supplement: Supplementary Information [file srep43871-s1.pdf]

Surveillance strategies for Classical Swine Fever in wild boar – a comprehensive evaluation study to ensure powerful surveillance

Katja Schulz, Marisa Peyre, Christoph Staubach, Birgit Schauer, Jana Schulz, Clémentine Calba, Barbara Häsler, Franz J. Conraths

```
### Simulation study - CSF in wild boar - alternative surveillance strategies ###
### 2015-10-26

### Part 1: Functions
## 1.1: Resample (see ?sample for more information)
resample <- function(x, ...) x[sample.int(length(x), ...)]

## 1.2: Free.from.disease
free.from.disease <-
  function(pop.size, design.prev, pos=0, se = 1, sp = 1, one.sided.conf = 0.95) {

    # if (pop.size > 1000) stop("Population size > 1000, Maximum population size =
    # 1000")
    if (design.prev < 0 | design.prev > 1) stop("Required: 0 <= design.prev <= 1")
    NI<-ceiling(pop.size*design.prev)
    if (pos > pop.size) stop("number of positives > population size")
    if (se < 0 | se > 1) stop("Required: 0 <= sensitivity <= 1")
    if (sp < 0 | sp > 1) stop("Required: 0 <= specificity <= 1")
    if (one.sided.conf < 0 | one.sided.conf > 1) stop("Required: 0 <= one sided
      confidence level <= 1")
    wIrrt <- 1-one.sided.conf
    free <- -50

    working.dir <- getwd()

    # set temporary directory
    sampling.dir=tempfile()
    sampling.dir=gsub("\\\\", .Platform$file.sep, sampling.dir)
    dir.create(sampling.dir)
    setwd(sampling.dir)

    write.table(c(se, sp, free, pos, pop.size, wIrrt, NI),
    file="Fkt_Parameter.txt", dec=".", row.names=FALSE, col.names=FALSE)

    file.dir = system.file("exec/nSeSpHypGeo.exe", package="exactSampling")
    file.dir

    if (file.exists(file.dir))
      system(shQuote(file.dir))
    else
    {setwd(working.dir)
      stop("wrong directory - .exe file not found")
    }

    result<-read.table("Fkt_Wert.txt", dec=".")

    file.remove("Fkt_Parameter.txt")
    file.remove("Fkt_Wert.txt")

    setwd(working.dir)
    unlink(sampling.dir)

    if (result[1,1]==-1) {stop("Problem cannot be solved")}
    else if (result[1,1]==0)
    {
```

```
      cat("    Exact    sample    size    to    test    freedom    from    disease",
        as.numeric(result[2,1]),"\n")
      cat(" Type two error: ",as.numeric(result[3,1]),"\n")}

invisible(list(min.sample=as.numeric(result[2,1]),error=as.numeric(result[3,1])))
  }


### Part 2: read in of data
## 2.1: RP related data
# District, Area, Index, Population
RP<-read.csv("RP_Population.csv", sep=';', dec=',', header=TRUE)

## 2.2: Hunting related data (RP)
# District, January, February, ..., November, December, FallWild
Hunt<-read.csv("RP_Hunt.csv", sep=';', dec=',', header=TRUE)
hunt<-rep(NA,length(Hunt[,1]))
for (i in 1:length(Hunt[,1])) {
  hunt[i]<-sum(Hunt[i,2:13])
}

## 2.3: Population structure
# Pop_structure, Pop_gen
Pop<-read.csv("RP_Popstructure.csv", sep=';', dec=',', header=TRUE)

## 2.4: Infection related data
infect<-read.csv("RP_Infection.csv", sep=';', dec=',', header=TRUE)
sero<-infect[,1]
viro<-infect[,2]

## 2.5: Surveillance data on monthly basis - data from NS
sur<-read.csv("NS_surv.csv", sep=';', dec=',', header=FALSE)

### Part 3: Simulation of population, infection and hunting
## 3.1 Simulation related parameters
# soiM = maximum value for start of infection (in month)
soiM=12
# SimRepM = Number of simulation repetitions
SimRepM=1000

for (soi in 1:soiM) {                   # soi = start.of.infection

  ## 3.1: Popopulation matrix
  pop.rp<-matrix(NA,nrow=sum(RP[,4]),ncol=8)
  #  number  of  rows  =  sum  of  the  estimated  population  size  of  the  24  considered
  # districts
  # number of columns: 8: 1 - Identification number
  #                       2 - Age         |
  #                            3 - Gender        >    values from population table
  #                                             RR+LR+frei
  #                       4 - Carcass     |
  #                       5 - District    -   estimated values
  #                       6 - Serology    \
  #                       7 - Virology    /   values from MVP
  #                        8 - Hunting      -   values from RR on district level and
  #                                               on monthly basis

  ## 3.2: Identification number (column 1)
  for (i in 1:sum(RP[,4])) {
    pop.rp[i,1]=i
  }

  ## 3.3: Age, gender and carcass (columns 2-4)
  #       values from RP_Popstructure_2015 04 12.csv
  #  Age: 1 - 0-1 year
  #       2 - 1-2 years
  #       3 - > 2 years
  #  Gender: 1 - female
  #          2 - male
  #  Carcass: 1 - shot dead
```

```r
#            2 - found dead
#            3 - shot sick
#            4 - RTA
if (Pop[1,2]>1) {
  for (i in 1:Pop[1,2]) {
    pop.rp[i,2]=1; pop.rp[i,3]=1; pop.rp[i,4]=1
  }
}
if (Pop[2,2]>(Pop[1,2]+1)) {
  for (i in (Pop[1,2]+1):Pop[2,2]) {
    pop.rp[i,2]=1; pop.rp[i,3]=1; pop.rp[i,4]=2
  }
}
if (Pop[3,2]>(Pop[2,2]+1)) {
  for (i in (Pop[2,2]+1):Pop[3,2]) {
    pop.rp[i,2]=1; pop.rp[i,3]=1; pop.rp[i,4]=3
  }
}
# ...
if (Pop[24,2]>(Pop[23,2]+1)) {
  for (i in (Pop[23,2]+1):Pop[24,2]) {
    pop.rp[i,2]=3; pop.rp[i,3]=2; pop.rp[i,4]=4
  }
}

## 3.4 District (population estimates) (column 5)
mem.kreis.new=NULL
for (l in 1:length(RP[,1])) {
  pop.mem<-pop.rp[which(!(pop.rp[,1] %in% mem.kreis.new)),1]
  mem.kreis<-resample(pop.mem,RP[l,4],replace=FALSE)
  mem.kreis.new<-c(mem.kreis,mem.kreis.new)
  for (i in 1:RP[l,4]) {
    pop.rp[mem.kreis[i],5]=l
  }
}

## 3.5 Serology (column 6)
#      values from MVP

sero.norm<-matrix(NA, nrow=2, ncol=3)
# Scaling factors for female individuals of the 3 age classes
sero.norm[1,]<-c(0.1202623, 0.2497499, 0.1340447)
# Scaling factors for male individuals of the 3 age classes
sero.norm[2,]<-c(0.1150383, 0.2164055, 0.1644993)

sero.prev.active<-array(NA, dim=c(3,2,12))
for (q in 1:3) {
  for (w in 1:2) {
    for (e in 1:12) {
      sero.prev.active[q,w,e]<-sero[e]*sero.norm[w,q]
    }
  }
}

dist<-rep(NA,SimRepM)
# Selection of the district in which the infection is set up
for (SimRep in 1:SimRepM) {
  cat(c('SimRep: ', SimRep, 'Soi: ', soi, '\n'))
  dist[SimRep]<-resample(1:length(RP[,1]),1,replace=FALSE)

  sero.pos.nr.active<-array(0,dim=c(3,2,soiM))
  pop.rp[,6]=0
  mem.sero.pos<-NULL
  len.mem<-length(pop.rp[which(pop.rp[,5]==dist[SimRep] & pop.rp[,4]==1),1])
  if (soi==1){
    for (e in soi:(soi+11)){
      for (q in 1:3){
        for (w in 1:2){
          sero.pos.nr.active[q,w,e]<-len.mem*sero.prev.active[q,w,e]
```

```
        if                  (round(sero.pos.nr.active[q,w,e])                  ==
            ceiling(sero.pos.nr.active[q,w,e])) {
              sero.pos.nr.active[q,w,e]<-ceiling(sero.pos.nr.active[q,w,e])
        }
        if                  (round(sero.pos.nr.active[q,w,e])                  !=
            ceiling(sero.pos.nr.active[q,w,e])) {
              sero.pos.nr.active[q,w,e]<-
                sample(c(round(sero.pos.nr.active[q,w,e]),
                  ceiling(sero.pos.nr.active[q,w,e])),        1,        prob=c(1-
                  (sero.pos.nr.active[q,w,e]-round(sero.pos.nr.active[q,w,e])),
                  sero.pos.nr.active[q,w,e]-round(sero.pos.nr.active[q,w,e])))
        }
      }
    }
    len.mem<-length(pop.rp[which(pop.rp[,5]==dist[SimRep]  &  pop.rp[,4]==1),1])-
              sum(sero.pos.nr.active)
    if (len.mem<0) {len.mem=0}
  }
}

if (soi>1) {
  for (e in soi:(soi+11)){
    for (q in 1:3){
      for (w in 1:2){
        if (e>=13) {
          sero.pos.nr.active[q,w,e%%13+1]<-len.mem*sero.prev.active[q,w,(e-
            soi+1)]
          if            (round(sero.pos.nr.active[q,w,(e%%13+1)])              ==
              ceiling(sero.pos.nr.active[q,w,(e%%13+1)])) {
                sero.pos.nr.active[q,w,(e%%13+1)]<-
                  ceiling(sero.pos.nr.active[q,w,(e%%13+1)])
          }
          if            (round(sero.pos.nr.active[q,w,(e%%13+1)])              !=
              ceiling(sero.pos.nr.active[q,w,(e%%13+1)])) {
                sero.pos.nr.active[q,w,(e%%13+1)]<-
                  sample(c(round(sero.pos.nr.active[q,w,(e%%13+1)]),
                  ceiling(sero.pos.nr.active[q,w,(e%%13+1)])),1,prob=c(1-
                  (sero.pos.nr.active[q,w,(e%%13+1)]-
                  round(sero.pos.nr.active[q,w,(e%%13+1)])),
                  sero.pos.nr.active[q,w,(e%%13+1)]                         -
                  round(sero.pos.nr.active[q,w,(e%%13+1)])))
          }
        } else {
          sero.pos.nr.active[q,w,e]<-len.mem*sero.prev.active[q,w,(e-soi+1)]
          if                 (round(sero.pos.nr.active[q,w,e])                 ==
              ceiling(sero.pos.nr.active[q,w,e])) {
                sero.pos.nr.active[q,w,e] <- ceiling(sero.pos.nr.active[q,w,e])
          }
          if                 (round(sero.pos.nr.active[q,w,e])                 !=
              ceiling(sero.pos.nr.active[q,w,e])) {
                sero.pos.nr.active[q,w,e]<-
                  sample(c(round(sero.pos.nr.active[q,w,e]),
                  ceiling(sero.pos.nr.active[q,w,e])),1,prob=c(1-
                  (sero.pos.nr.active[q,w,e]-
                  round(sero.pos.nr.active[q,w,e])),sero.pos.nr.active[q,w,e]-
                  round(sero.pos.nr.active[q,w,e])))
          }
        }
      }
    }
    len.mem<-length(pop.rp[which(pop.rp[,5]==dist[SimRep]  &  pop.rp[,4]==1),1])-
                   sum(sero.pos.nr.active)
    if (len.mem<0) {len.mem=0}
  }
}

for (q in 1:3){
  for (w in 1:2){
    for (e in soi:(soi+11)){
      if (e>=13) {
```

```
          if     (length(pop.rp[which(pop.rp[,2]==q     &     pop.rp[,3]==w     &
              pop.rp[,4]==1  &  pop.rp[,6]==0  &  pop.rp[,5]==dist[SimRep]),1])  >=
              sero.pos.nr.active[q,w,(e%%13+1)]]){
                mem.sero.pos<-resample(pop.rp[which(pop.rp[,2]==q  &  pop.rp[,3]==w
                  & pop.rp[,4]==1  & pop.rp[,6]==0  & pop.rp[,5]==dist[SimRep]),1],
                  sero.pos.nr.active[q,w,(e%%13+1)], replace=FALSE)
                pop.rp[mem.sero.pos,6]<-e
          }
          if  (length(pop.rp[which(pop.rp[,2]==q & pop.rp[,3]==w & pop.rp[,4]==1  &
              pop.rp[,6]==0         &         pop.rp[,5]==dist[SimRep]),1])         <
              sero.pos.nr.active[q,w,(e%%13+1)]]){
                mem.sero.pos<-pop.rp[which(pop.rp[,2]==q    &    pop.rp[,3]==w    &
                  pop.rp[,4]==1 & pop.rp[,6]==0 & pop.rp[,5]==dist[SimRep]),1]
                pop.rp[mem.sero.pos,6]<-e
          }
        } else {
          if  (length(pop.rp[which(pop.rp[,2]==q & pop.rp[,3]==w & pop.rp[,4]==1  &
              pop.rp[,6]==0         &        pop.rp[,5]==dist[SimRep]),1])         >=
              sero.pos.nr.active[q,w,e]){
                mem.sero.pos<-resample(pop.rp[which(pop.rp[,2]==q  &  pop.rp[,3]==w
                  & pop.rp[,4]==1  & pop.rp[,6]==0  & pop.rp[,5]==dist[SimRep]),1],
                  sero.pos.nr.active[q,w,e],replace=FALSE)
                pop.rp[mem.sero.pos,6]<-e
          }
          if  (length(pop.rp[which(pop.rp[,2]==q & pop.rp[,3]==w & pop.rp[,4]==1  &
              pop.rp[,6]==0         &         pop.rp[,5]==dist[SimRep]),1])         <
              sero.pos.nr.active[q,w,e]){
                mem.sero.pos<-pop.rp[which(pop.rp[,2]==q    &    pop.rp[,3]==w    &
                  pop.rp[,4]==1 & pop.rp[,6]==0 & pop.rp[,5]==dist[SimRep]),1]
                pop.rp[mem.sero.pos,6]<-e
          }
        }
      }
    }
  }

## 3.6: Virology (column 7)
#       values from MVP
#       for animals marked for active surveillance (pop.rp[,4]==1)
pop.rp[,7]=0

for (t in 1:length(pop.rp[,1])) {
  if (pop.rp[t,6]==1) {pop.rp[t,7]=-9}
  for (z in 2:25) {
    if (pop.rp[t,6]==z) {
      pop.rp[t,7]=z-1
    }
  }
}

#     Virology     for     animals     marked     for     passive     surveillance
#       (pop.rp[,4] %in% c(2,3,4))
viro.pos.nr.passive<-rep(0,soiM)
len.mem<-length(pop.rp[which(pop.rp[,5]==dist[SimRep]     &     pop.rp[,4]     %in%
                c(2,3,4)),1])
for (e in soi:(soi+11)){
  if (e>=13) {
    viro.pos.nr.passive[e%%13+1]<-len.mem*viro[e%%13+1]
    if                  (round(viro.pos.nr.passive[e%%13+1])                  ==
        ceiling(viro.pos.nr.passive[e%%13+1])) {
          viro.pos.nr.passive[e%%13+1]<-ceiling(viro.pos.nr.passive[e%%13+1])
    }
    if                  (round(viro.pos.nr.passive[e%%13+1])                  !=
        ceiling(viro.pos.nr.passive[e%%13+1])) {
          viro.pos.nr.passive[e%%13+1]<-
            sample(c(round(viro.pos.nr.passive[e%%13+1]),
              ceiling(viro.pos.nr.passive[e%%13+1])),        1,        prob=c(1-
              (viro.pos.nr.passive[e%%13+1]-
              round(viro.pos.nr.passive[e%%13+1])),
```

```
              viro.pos.nr.passive[e%%13+1]                             -
              round(viro.pos.nr.passive[e%%13+1])))
    }
  } else {
    viro.pos.nr.passive[e]<-len.mem*viro[e]
    if (round(viro.pos.nr.passive[e])==ceiling(viro.pos.nr.passive[e])) {
      viro.pos.nr.passive[e]<-ceiling(viro.pos.nr.passive[e])
    }
    if (round(viro.pos.nr.passive[e])!=ceiling(viro.pos.nr.passive[e])) {
      viro.pos.nr.passive[e]<-sample(c(round(viro.pos.nr.passive[e]),
        ceiling(viro.pos.nr.passive[e])),  1,  prob=c(1-(viro.pos.nr.passive[e]-
        round(viro.pos.nr.passive[e])),viro.pos.nr.passive[e]-
        round(viro.pos.nr.passive[e])))
    }
  }
}

for (e in soi:(soi+11)){
  if (e>=13) {
    if   (length(pop.rp[which(pop.rp[,4]   %in%   c(2,3,4)   &   pop.rp[,7]==0   &
        pop.rp[,5]==dist[SimRep]),1]) >= viro.pos.nr.passive[e%%13+1]){
          mem.viro.pos<-resample(pop.rp[which(pop.rp[,4]    %in%    c(2,3,4)    &
            pop.rp[,7]==0              &             pop.rp[,5]==dist[SimRep]),1],
            viro.pos.nr.passive[e%%13+1], replace=FALSE)
      pop.rp[mem.viro.pos,7]<-e
    }
    if   (length(pop.rp[which(pop.rp[,4]   %in%   c(2,3,4)   &   pop.rp[,7]==0   &
        pop.rp[,5]==dist[SimRep]),1]) < viro.pos.nr.passive[e%%13+1]){
          mem.viro.pos<-pop.rp[which(pop.rp[,4] %in% c(2,3,4) & pop.rp[,7]==0 &
            pop.rp[,5]==dist[SimRep]),1]
          pop.rp[mem.viro.pos,7]<-e
    }
  } else {
    if   (length(pop.rp[which(pop.rp[,4]   %in%   c(2,3,4)   &   pop.rp[,7]==0   &
        pop.rp[,5]==dist[SimRep]),1])>=viro.pos.nr.passive[e]){
          mem.viro.pos<-resample(pop.rp[which(pop.rp[,4]    %in%    c(2,3,4)    &
            pop.rp[,7]==0              &             pop.rp[,5]==dist[SimRep]),1],
            viro.pos.nr.passive[e],replace=FALSE)
          pop.rp[mem.viro.pos,7]<-e
    }
    if   (length(pop.rp[which(pop.rp[,4]   %in%   c(2,3,4)   &   pop.rp[,7]==0   &
        pop.rp[,5]==dist[SimRep]),1])<viro.pos.nr.passive[e]){
          mem.viro.pos<-pop.rp[which(pop.rp[,4] %in% c(2,3,4) & pop.rp[,7]==0 &
            pop.rp[,5]==dist[SimRep]),1]
          pop.rp[mem.viro.pos,7]<-e
    }
  }
}

## 3.7: Surveillance (column 8)
#       active: shot dead
#       passive: found dead, shot sick, RTA
active<-rep(as.numeric(Hunt[dist[SimRep],2:13]),2)
passive<-rep(as.numeric(Hunt[dist[SimRep],14])/12,24)
for (pa in 1:24) {
  if (round(passive[pa])==ceiling(passive[pa])) {
    passive[pa]=ceiling(passive[pa])
  } else {
    passive[pa]<-sample(c(round(passive[pa]),ceiling(passive[pa])),           1,
                 prob=c(1-(passive[pa]-round(passive[pa])),         passive[pa]-
                 round(passive[pa])))
  }
}

active_sim<-array(NA,dim=c(length(seq(soi,soi+11,1)),max(active)))
passive_sim<-array(NA,dim=c(length(seq(soi,soi+11,1)),max(passive)))

pop.rp[,8]<-0
for (i in soi:(soi+11)) {
```

```r
  if    (length(pop.rp[which(pop.rp[,8]==0    &    pop.rp[,5]==dist[SimRep]    &
      pop.rp[,4]==1),1])>=active[i]) {
        mem_active<-resample(pop.rp[which(pop.rp[,8]==0                          &
          pop.rp[,5]==dist[SimRep] & pop.rp[,4]==1),1],active[i],replace=FALSE)
  }
  if    ((length(pop.rp[which(pop.rp[,8]==0    &    pop.rp[,5]==dist[SimRep]    &
      pop.rp[,4]==1),1])<active[i])    &    (length(pop.rp[which(pop.rp[,8]==0    &
      pop.rp[,5]==dist[SimRep] & pop.rp[,4]==1),1])>0)) {
        mem_active<-pop.rp[which(pop.rp[,8]==0   &   pop.rp[,5]==dist[SimRep]   &
          pop.rp[,4]==1),1]
  }
  if    (length(pop.rp[which(pop.rp[,8]==0    &    pop.rp[,5]==dist[SimRep]    &
      pop.rp[,4]==1),1])==0) {
        mem_active<-NULL
  }
  pop.rp[which(pop.rp[,1] %in% mem_active),8]=i


  if (length(pop.rp[which(pop.rp[,8]==0 & pop.rp[,5]==dist[SimRep] & pop.rp[,4]
      %in% c(2,3,4)),1]) >= passive[i]) {
        mem_passive<-resample(pop.rp[which(pop.rp[,8]==0                         &
          pop.rp[,5]==dist[SimRep]  & pop.rp[,4]  %in%  c(2,3,4)),1],  passive[i],
          replace=FALSE)
  }
  if    ((length(pop.rp[which(pop.rp[,8]==0    &    pop.rp[,5]==dist[SimRep]    &
      pop.rp[,4]            %in%            c(2,3,4)),1])<passive[i])            &
      (length(pop.rp[which(pop.rp[,8]==0     &     pop.rp[,5]==dist[SimRep]     &
      pop.rp[,4] %in% c(2,3,4)),1])>0)) {
        mem_passive<-pop.rp[which(pop.rp[,8]==0   &   pop.rp[,5]==dist[SimRep]   &
          pop.rp[,4] %in% c(2,3,4)),1]
  }
  if (length(pop.rp[which(pop.rp[,8]==0 & pop.rp[,5]==dist[SimRep] & pop.rp[,4]
      %in% c(2,3,4)),1])==0) {
        mem_passive<-NULL
  }
  pop.rp[which(pop.rp[,1] %in% mem_passive),8]=i


  if (length(mem_active)>0){
    if (active[i]>0) {
      for (j in 1:active[i]) {
        if (i>=13) {
          active_sim[(i%%13+1),j]<-mem_active[j]
        } else {
          active_sim[i,j]<-mem_active[j]
        }
      }
    }
  }

  if (length(mem_passive)>0){
    if (passive[i]>0) {
      for (j in 1:passive[i]) {
        if (i>=13) {
          passive_sim[(i%%13+1),j]<-mem_passive[j]
        } else {
          passive_sim[i,j]<-mem_passive[j]
        }
      }
    }
  }
}

### 3.8: Saving of relevant data
pop.rp.new<-pop.rp[which(pop.rp[,5]==dist[SimRep]),]

file            =            paste(paste(paste(paste("RP_soi=",            soi,
       sep=''),'_SimRep=',sep=''),SimRep,sep=''),".Rdata",sep='')

save(pop.rp.new, active_sim, passive_sim, file=file)
```

```
    }
  }

  ### Part 4: Surveillance scenarios (example code)
  ## 4.1a: 59 samples out of hunting bag RR - active surveillance
  cat('Surveillance scenario 1a \n')
  active.year<-array(NA,c(soiM,SimRepM,max(hunt)))
  for (soi in 1:soiM) {
    for (SimRep in 1:SimRepM) {
      file=paste(paste(paste(paste("RP_soi=",   soi,   sep=''),   '_SimRep=',sep=''),
           SimRep,sep=''), ".Rdata",sep='')
      load(file=file)
      if (length(c(active_sim[1,which(is.na(active_sim[1,])==FALSE)],
                   active_sim[2,which(is.na(active_sim[2,])==FALSE)],
                   active_sim[3,which(is.na(active_sim[3,])==FALSE)],
                   active_sim[4,which(is.na(active_sim[4,])==FALSE)],
                   active_sim[5,which(is.na(active_sim[5,])==FALSE)],
                   active_sim[6,which(is.na(active_sim[6,])==FALSE)],
                   active_sim[7,which(is.na(active_sim[7,])==FALSE)],
                   active_sim[8,which(is.na(active_sim[8,])==FALSE)],
                   active_sim[9,which(is.na(active_sim[9,])==FALSE)],
                   active_sim[10,which(is.na(active_sim[10,])==FALSE)],
                   active_sim[11,which(is.na(active_sim[11,])==FALSE)],
                   active_sim[12,which(is.na(active_sim[12,])==FALSE)]))>0) {
  active.year[soi,SimRep,
    1:(length(c(active_sim[1,which(is.na(active_sim[1,])==FALSE)],
                active_sim[2,which(is.na(active_sim[2,])==FALSE)],
                active_sim[3,which(is.na(active_sim[3,])==FALSE)],
                active_sim[4,which(is.na(active_sim[4,])==FALSE)],
                active_sim[5,which(is.na(active_sim[5,])==FALSE)],
                active_sim[6,which(is.na(active_sim[6,])==FALSE)],
                active_sim[7,which(is.na(active_sim[7,])==FALSE)],
                active_sim[8,which(is.na(active_sim[8,])==FALSE)],
                active_sim[9,which(is.na(active_sim[9,])==FALSE)],
                active_sim[10,which(is.na(active_sim[10,])==FALSE)],
                active_sim[11,which(is.na(active_sim[11,])==FALSE)],
                active_sim[12,which(is.na(active_sim[12,])==FALSE)])))]<-
  c(active_sim[1,which(is.na(active_sim[1,])==FALSE)],
    active_sim[2,which(is.na(active_sim[2,])==FALSE)],
    active_sim[3,which(is.na(active_sim[3,])==FALSE)],
    active_sim[4,which(is.na(active_sim[4,])==FALSE)],
    active_sim[5,which(is.na(active_sim[5,])==FALSE)],
    active_sim[6,which(is.na(active_sim[6,])==FALSE)],
    active_sim[7,which(is.na(active_sim[7,])==FALSE)],
    active_sim[8,which(is.na(active_sim[8,])==FALSE)],
    active_sim[9,which(is.na(active_sim[9,])==FALSE)],
    active_sim[10,which(is.na(active_sim[10,])==FALSE)],
    active_sim[11,which(is.na(active_sim[11,])==FALSE)],
    active_sim[12,which(is.na(active_sim[12,])==FALSE)])
      }
    }
  }

  active_sim.59<-array(NA,dim=c(soiM,SimRepM,59))
  for (soi in 1:soiM) {
    for (k in 1:SimRepM) {
      if (length(active.year[soi,k,which(is.na(active.year[soi,k,])==FALSE)])>59) {
        active_sim.59[soi,k,]<-resample(active.year[soi,                          k,
          which(is.na(active.year[soi,k,])==FALSE)], 59, replace=FALSE)
      }
      if   (length(active.year[soi,k,which(is.na(active.year[soi,k,])==FALSE)])<=59   &
          length(active.year[soi,k,which(is.na(active.year[soi,k,])==FALSE)])>0) {
        active_sim.59[soi,        k,         (1:length(active.year[soi,          k,
          which(is.na(active.year[soi,k,])==FALSE)]))]<-
          active.year[soi,k,which(is.na(active.year[soi,k,])==FALSE)]
      }
    }
  }

  # 4.1a: serological examination
```

```
time.59<-array(NA,c(soiM,12,SimRepM))
for (soi in 1:soiM) {
  for (k in 1:SimRepM) {
    file=paste(paste(paste(paste("RP_soi=", soi, sep=''), '_SimRep=', sep=''), k,
         sep=''), ".Rdata", sep='')
    load(file=file)
    for (i in soi:(soi+11)) {
      if  (length(pop.rp.new[which(pop.rp.new[,1]  %in%  active_sim.59[soi,k,]  &
          pop.rp.new[,6]!=0 & pop.rp.new[,6]<=i & pop.rp.new[,8]==i),6]>0)) {
        if (i>=13){
          time.59[soi,(i%%13+1),k]<-1
        } else {
          time.59[soi,i,k]<-1
        }
      }
    }
  }
}

time.59.<-array(NA,c(soiM,SimRepM))
for (soi in 1:soiM) {
  for (k in 1:SimRepM) {
    for (i in soi:(soi+11)) {
      if (i>=13) {
        if (is.na(time.59[soi,(i%%13+1),k])==FALSE) {
          time.59.[soi,k]=min(i,time.59.[soi,k],na.rm=TRUE)
        }
      } else {
        if (is.na(time.59[soi,i,k])==FALSE) {
          time.59.[soi,k]=min(i,time.59.[soi,k],na.rm=TRUE)
        }
      }
    }
  }
}
time.59.[is.na(time.59.)==TRUE]=0

counts.59<-array(NA,c(13,soiM))
for (soi in 1:soiM) {
  for (i in soi:(soi+11)) {
    counts.mem<-hist(time.59.[soi,],breaks = seq(-1,(soi+11),1))$counts
    counts.59[1,soi]<-counts.mem[1]
    counts.59[(2:13),soi]<-counts.mem[(soi+1):(soi+12)]
  }
}

## 4.1b: 59 out of hunting bag - on monthly basis (data from NS)
cat('Surveillance scenario 1b \n')
sur59<-resample(seq(1,12,1),59, replace=TRUE, prob=sur[1,2:13])
nr.sur59<-rep(0,12)
for (t in 1:12) {
  nr.sur59[t]<-length(sur59[which(sur59==t)])
}

active_sim.59m<-array(NA,c(soiM,SimRepM,12,max(nr.sur59)))
for (soi in 1:soiM) {
  for (SimRep in 1:SimRepM) {
    file=paste(paste(paste(paste("RP_soi=",  soi,  sep=''),  '_SimRep=',  sep=''),
         SimRep, sep=''), ".Rdata", sep='')
    load(file=file)
    for (i in soi:(soi+11)) {
      if (i>=13) {
        if (nr.sur59[i%%13+1]>0) {
          if  (length(active_sim[i%%13+1,which(is.na(active_sim[i%%13+1,])==FALSE)])
              > nr.sur59[i%%13+1]){
            active_sim.59m[soi,SimRep,i%%13+1,(1:nr.sur59[i%%13+1])]<-
              resample(active_sim[i%%13+1, which(is.na(active_sim[i%%13+1,]) ==
              FALSE)], nr.sur59[i%%13+1], replace=FALSE)
          }
```

```
          } else if ((length(active_sim[i%%13+1, which(is.na(active_sim[i%%13+1,]) ==
                     FALSE)]) < nr.sur59[i%%13+1]) & (length(active_sim[i%%13+1,
                     which(is.na(active_sim[i%%13+1,]) == FALSE)]) > 0)){
                       active_sim.59m[soi, SimRep, i%%13+1,
                       (1:length(active_sim[i%%13+1,
                       which(is.na(active_sim[i%%13+1,]) == FALSE)]))]<-
                       active_sim[i%%13+1, which(is.na(active_sim[i%%13+1,]) ==
                       FALSE)]
          }
        } else {
          if (nr.sur59[i]>0) {
            if (length(active_sim[i, which(is.na(active_sim[i,])==FALSE)]) >
                nr.sur59[i]){
                  active_sim.59m[soi,SimRep,i,(1:nr.sur59[i])]<-
                    resample(active_sim[i, which(is.na(active_sim[i,]) == FALSE)],
                    nr.sur59[i], replace=FALSE)
            }
          } else if (length(active_sim[i,which(is.na(active_sim[i,])==FALSE)]) <
                     nr.sur59[i] & (length(active_sim[i, which(is.na(active_sim[i,])
                     == FALSE)])>0)) {
                       active_sim.59m[soi, SimRep, i, (1:length(active_sim[i,
                       which(is.na(active_sim[i,]) == FALSE)]))]<-
                       active_sim[i,which(is.na(active_sim[i,])==FALSE)]
          }
        }
      }
    }
}

# 4.1b: serological examination
time.59m<-array(NA,c(soiM,12,SimRepM))
for (soi in 1:soiM) {
  for (k in 1:SimRepM) {
    file=paste(paste(paste(paste("RP_soi=", soi, sep=''), '_SimRep=', sep=''), k,
         sep=''), ".Rdata",sep='')
    load(file=file)
    for (i in soi:(soi+11)) {
      if (i>=13){
        if (length(pop.rp.new[which(pop.rp.new[,1] %in% active_sim.59m[soi, k,
            i%%13+1, which(is.na(active_sim.59m[soi, k, i%%13+1,])==FALSE)] &
            pop.rp.new[,6]!=0 & pop.rp.new[,6]<=i & pop.rp.new[,8]==i),6]>0)) {
              time.59m[soi,(i%%13+1),k]<-1
        }
      } else {
        if (length(pop.rp.new[which(pop.rp.new[,1] %in% active_sim.59m[soi, k, i,
            which(is.na(active_sim.59m[soi,k,i,])==FALSE)] & pop.rp.new[,6]!=0 &
            pop.rp.new[,6]<=i & pop.rp.new[,8]==i),6]>0)) {
              time.59m[soi,i,k]<-1
        }
      }
    }
  }
}

time.59m.<-array(NA,c(soiM,SimRepM))
for (soi in 1:soiM) {
  for (k in 1:SimRepM) {
    for (i in soi:(soi+11)) {
      if (i>=13) {
        if (is.na(time.59m[soi,(i%%13+1),k])==FALSE) {
          time.59m.[soi,k]=min(i,time.59m.[soi,k],na.rm=TRUE)
        }
      } else {
        if (is.na(time.59m[soi,i,k])==FALSE) {
          time.59m.[soi,k]=min(i,time.59m.[soi,k],na.rm=TRUE)
        }
      }
    }
  }
}
```

```
time.59m.[is.na(time.59m.)==TRUE]=0

counts.59m<-array(NA,c(13,soiM))
for (soi in 1:soiM) {
  for (i in soi:(soi+11)) {
    counts.mem<-hist(time.59m.[soi,],breaks = seq(-1,(soi+11),1))$counts
    counts.59m[1,soi]<-counts.mem[1]
    counts.59m[(2:13),soi]<-counts.mem[(soi+1):(soi+12)]
  }
}

### Part 5: Results
## 5.1 Saving results
save(counts.59, counts.59m, file="surveillance_Standard.Rdata")

## 5.2 Generating table containing number of simulation runs that did not detect
the infection (example code)
counts.final<-matrix(NA, nrow=2, ncol=12)
counts.final[1,]<-counts.59[1,]
counts.final[2,]<-counts.59m[1,]

write.csv(counts.final,file="RP_Standard - not_found_new.csv")

## 5.3 Timeliness (example code)
timeliness<-rep(NA,2)
weight<-seq(12,1,-1)

counts.59.pc<-matrix(NA, ncol=12, nrow=12)
for (i in 2:13) {
  for (j in 1:12) {
    counts.59.pc[i-1,j]<-counts.59[i,j]/sum(counts.59[2:13,j])
  }
}
rowavg.counts.59<-rep(NA,12)
for (i in 1:12) {
  rowavg.counts.59[i]<-sum(counts.59.pc[i,])/12
}
timeliness[1]<-sum(weight*rowavg.counts.59)/78

counts.59m.pc<-matrix(NA, ncol=12, nrow=12)
for (i in 2:13) {
  for (j in 1:12) {
    counts.59m.pc[i-1,j]<-counts.59m[i,j]/sum(counts.59m[2:13,j])
  }
}
rowavg.counts.59m<-rep(NA,12)
for (i in 1:12) {
  rowavg.counts.59m[i]<-sum(counts.59m.pc[i,])/12
}
timeliness[2]<-sum(weight*rowavg.counts.59m)/78

## 5.6 Generating figures (example code)
bmp("counts.59.pc.bmp")
filled.contour(x=seq(1,soiM,1), y=seq(1,12,1), t(counts.59.pc), col =
rainbow(100,start=0,end=1),
nlevels=max(counts.59.pc)*100,xlim=c(1,12),ylim=c(1,12))
dev.off()

bmp("counts.59m.pc.bmp")
filled.contour(x=seq(1,soiM,1), y=seq(1,12,1), t(counts.59m.pc), col =
rainbow(100,start=0,end=1),
nlevels=max(counts.59m.pc)*100,xlim=c(1,12),ylim=c(1,12))
```
